# Selenium Deficiency in Lymphedema and Lipedema—A Retrospective Cross-Sectional Study from a Specialized Clinic

**DOI:** 10.3390/nu12051211

**Published:** 2020-04-25

**Authors:** Christina Pfister, Horst Dawczynski, Franz-Josef Schingale

**Affiliations:** 1 Biosyn Arzneimittel GmbH, Schorndorfer Straße 32, 70734 Fellbach, Germany; horst_dawczynski@biosyn.de; 2 Lympho Opt Fachklinik, Happurger Straße 15, 91224 Hohenstadt, Germany; franz-josef.schingale@lympho-opt.de

**Keywords:** selenium, lymphedema, lipedema, obesity, oxidative stress, inflammation

## Abstract

Background: Selenium is a trace element, which is utilized by the human body in selenoproteins. Their main function is to reduce oxidative stress, which plays an important role in lymphedema and lipedema. In addition, selenium deficiency is associated with an impaired immune function. The aim of this study was to determine the prevalence of selenium deficiency in these conditions, and if it is associated with disease severity and an associated medical condition such as obesity. Methods: This cross-sectional study is an anonymized, retrospective analysis of clinical data that was routinely recorded in a clinic specialized in lymphology. The data was comprised from 791 patients during 2012–2019, in which the selenium status was determined as part of their treatment. Results: Selenium deficiency proved common in patients with lymphedema, lipedema, and lipo-lymphedema affecting 47.5% of the study population. Selenium levels were significantly lower in patients with obesity-related lymphedema compared to patients with cancer-related lymphedema (96.6 ± 18.0 μg/L vs. 105.1 ± 20.2 μg/L; *p* < 0.0001). Obesity was a risk factor for selenium deficiency in lymphedema (OR 2.19; 95% CI 1.49 to 3.21), but not in lipedema. Conclusions: In countries with low selenium supply, selenium deficiency is common, especially in lymphedema patients. Therefore, it would be sensible to check the selenium status in lymphedema patients, especially those with obesity, as the infection risk of lymphedema is already increased.

## 1. Introduction

Clinics specialized in lymphological diseases manage not only patients with cancer treatment-related secondary lymphedema, but also treat patients with primary lymphedema, as well as secondary lymphedema due to obesity, lipedema, or lipo-lymphedema. The pathogenesis of lymphedema is a progressive process, consisting of lymphatic leakage and stagnation, chronic inflammation, adipose tissue expansion and fibrosis [1]. In contrast to lymphedema, lipedema is characterized by bilaterally increased circumference of extremities, pain sensation, and bruising [2]. Lipedema leaves most distal parts of the body, i.e., hands and feet, unaffected. In early stages, lipedema shows increased lymphatic flow, not lymphatic insufficiency [3]. The tissue water content in lipedema patients is in the range of healthy controls [4]. However, patients with lipedema may develop secondary lymphedema, if the fatty deposits compromise the lymphatic system [5]. The combination of lipedema and lymphatic impairment is called lipo-lymphedema [6].

Secondary lymphedema is mostly associated with cancer treatment as common and debilitating progressive sequelae. Recently, another reason has come to the fore: obesity-related lymphatic impairment [7]. Obesity is usually defined as a body mass index (BMI) ≥ 30 kg/m^2^. In lipedema, obesity is proving to be the most common comorbidity. Up to 80% of lipedema patients are obese [8]. Obese patients with lipedema are at risk for obesity-associated lymphedema [2].

Obesity appears to be associated with selenium deficiency [9,10,11]. A reduced selenium status is significantly associated with a BMI ≥ 30 kg/m^2^ in women [10,11]. Alasfar et al. showed that morbidly obese patients (BMI ≥ 40 kg/m^2^) display significantly reduced serum selenium concentrations [12]. Significantly reduced selenium status in obesity appears to result from obesity-related oxidative stress [13]. Obesity is moreover associated with a state of chronic inflammation, which also contributes to the pro-oxidant environment of the condition [14].

Selenium plays an important role in inflammation and immunity [15]. Selenium deficiency negatively affects immune cells during activation, differentiation, and proliferation. This is related to increased oxidative stress. Functions like protein folding and calcium flux in immune cells may also be impaired under selenium deficient conditions [15]. Erysipelas are frequent infectious complications of lymphedema [16]. Kasseroller et al. showed that high-dose sodium selenite—an inorganic selenium form—can reduce the incidence of erysipelas [17]. Selenium status was not determined in this study or in other comparable trials [18,19,20]. Therefore, it is an open question; if selenium deficiency may be problematic, sufficiency could be protective or lymphedema treatment with high-dosed sodium selenite could be independent of selenium status.

In obesity, lymphatic function in adipose tissue and drainage capacity in the lower extremities seems to be reduced [21]. In the tissue of chronic lymphedema patients, the formation of reactive oxygen species (ROS) is enhanced and lipid peroxidation processes are accelerated [22]. Siems et al. additionally showed that reduced glutathione (GSH) concentrations in blood were decreased in chronic lymphedema patients and glutathione disulfide (oxidized glutathione; GSSG) was elevated, resulting in a three-fold higher glutathione ratio, an indicator for oxidative stress [22]. The authors concluded that the oxidative stress related changes are a consequence of the lymphedema, since the control group that had treated tumors but no lymphedema had data comparable to the healthy controls. In lipedema patients, Siems et al. showed that serum concentrations of malondialdehyde (MDA) and plasma protein carbonyls were increased compared to healthy controls [23]. Therefore, oxidative stress also plays an important role in lipedema.

The functional gene expression analysis of a mouse model of acute acquired lymphedema showed that genes involved in the immune response, stress response, and complement activation were induced in lymphedema tissue [24]. These included several, mostly stress-responsive, selenoproteins, i.e., glutathione peroxidase 1. These selenoproteins are highly dependent on an adequate selenium supply. Glutathione peroxidase 1 reaches maximal activity at plasma selenium concentrations of 95 μg/L [25]. In selenium deficiency, glutathione peroxidase 1 is depleted in many organs [26]. The selenium transport protein, selenoprotein P, does not reach optimal activity in the plasma until 124 μg/L selenium [27]. Mean serum selenium levels in Germany are much lower than in other countries, with values of 74.3 (95% CI 69.8–79.0) μg/L registered in men and 73.2 (95% CI 67.4–79.6) μg/L in women [28]. Muecke et al. determined whole blood selenium concentrations in healthy males. Mean selenium value was 74.1 ± 23.0 μg/L. The selenium range was 43.6 to 127.0 μg/L [29], probably due to low selenium intake (34–60 μg selenium per day) [30]. The reference values for selenium intake in Germany for adults are 70 μg for men and 60 μg for women [31].

The aim of this observational study was to determine the prevalence of selenium deficiency in patients with primary or secondary lymphedema, lipedema, or lipo-lymphedema. In addition, the influence of obesity on the selenium status in these patients was evaluated.

## 2. Materials and Methods

### 2.1. Study Design

An ethics committee vote was not necessary, because clinic intern register data were used in this retrospective cross-sectional study. The data was raised routinely in patient care. Written informed consents for the data analysis of patient data were obtained from all participating patients in accordance with the Declaration of Helsinki. The clinical data were recorded in a clinic specialized in lymphology (Lympho-Opt Clinic Pommelsbrunn-Hohenstadt, Germany). The data of patients treated for lymphedema, lipedema or lipo-lymphedema, in which the selenium status was determined as part of their treatment, were anonymized for two time periods: from 2012 to 2016 (*n* = 236) and from 2018 to 2019 (*n* = 555). The anonymized data included gender, diagnosis, BMI, and selenium concentrations in whole blood. Diagnosis was determined by FJS based on the German S2K guidelines, which included positive Stemmer’s test, sonography or indocyanine green (ICG) fluorescence lymphography [32]. Overweight and obesity were classified according to BMI (overweight 25 to 29.9 kg/m^2^ and obesity ≥30 kg/m^2^).

### 2.2. Measurement of Whole Blood Selenium

Whole blood selenium samples were obtained at the beginning of the rehabilitation stay at the Lympho-Opt clinic, using tubes for trace elements/metal analytic. Blood samples were sent to a certified laboratory (biosyn Arzneimittel GmbH, Fellbach, Germany). Selenium levels were measured by microwave digestion and flameless atomic absorption spectrometry, according to the method of Winnefeld et al. [33] Selenium deficiency was assessed using the reference range defined by German authorities [34]. Selenium values in whole blood < 100 μg/L and < 80 μg/L in serum are defined as deficient.

### 2.3. Statistical Analysis

All data were stored and analyzed using GraphPad 8.3. All continuous data are presented as means ± standard deviation (SD), and the differences were assessed by one-way analysis of variance (normal distribution) or Kruskal–Wallis H test (non-normal distribution). All categorical data are presented as percentages; the differences were assessed by Pearson chi-square test. Differences between whole blood selenium concentrations in continuous variables were analyzed by Student’s t test (normal distribution) and Mann–Whitney test (non-normal distribution) for independent samples. One-way ANOVA was used to compare whole blood selenium concentrations in three or more groups (*p* trend). Needed sample size was calculated using G × Power 3.1.9.7. To calculate the difference between two independent means (two groups), α = 0.05 and power = 0.80 was used. Effect size was calculated using Hedges’ g, as the sample size of each group was not the same. All *p* values were 2-sided statistical tests and were considered statistically significant if < 0.05.

## 3. Results

### 3.1. Patients Characteristics

The study included two time periods from 2012 to 2016 (*n* = 236) and from 2018 to 2019 (*n* = 555). Three hundred and forty-seven of 791 patients were diagnosed with a secondary lymphedema (Table 1). In 146 of 347 patients, secondary lymphedema was a sequelae of cancer treatment. Most participants were women (*n* = 676, 85.5%). Lymphedema was the most common diagnosis in the male patients (106 of 115). There were not any age- or sex-dependent effects on whole blood selenium concentration (data not shown). The study population was stratified for body mass index (BMI) into three groups: < 30 (normal weight and overweight); ≥ 30 < 40 (obese), and ≥ 40 (morbidly obese). Most lymphedema patients had a BMI < 30 (54.8 %; 234/427)). In contrast, patients with lipedema and lipo-lymphedema were mostly obese (74.8%, respectively 75.6%). Baseline characteristics of the study population, stratified for BMI, are presented in Table S1.

### 3.2. Selenium Deficiency in the Overall Study Population

The mean selenium concentration in whole blood was 100.6 ± 17.4 μg/L (Table 1). Selenium deficiency affected nearly half of the study population (47.5%), with significant differences in prevalence between lipedema, primary lymphedema, secondary lymphedema and lipo-lymphedema (41.9% < 43.6% < 49.0% < 53.0%; *p* = 0.0002).

In addition, there was a significant difference between selenium levels as a function of BMI. The mean selenium concentration in whole blood was significantly lower in obese and morbidly obese patients compared to those with BMI < 30 (*p* < 0.0001) (Table S1). The risk of selenium deficiency was 1.7-fold higher in patients with BMI ≥ 30 (OR 1.73; 95% CI 1.30 to 2.30).

### 3.3. Selenium Deficiency in Lymphedema

**Figure 1:**
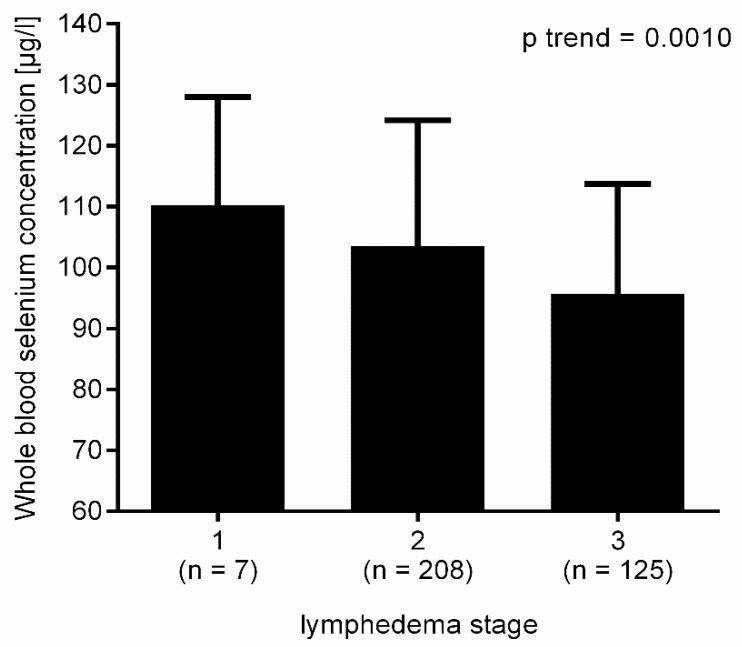
Decreasing selenium values with increasing lymphedema stage.

Whole blood selenium levels did not significantly differ between patients with primary and secondary lymphedema (*p* = 0.1827) (Table 2). Whole blood selenium concentration by lymphedema stage was analyzed combined for primary and secondary lymphedema. Selenium levels decreased with increasing lymphedema stage (*p* trend = 0.0008; Figure 1). As the number of lymphedema stage I was very small (*n* = 7), the risk for selenium deficiency was compared between lymphedema stage II and III. Patients with lymphedema stage III had a 2.2-fold higher risk for selenium deficiency. (OR 2.19; 95% CI 1.40 to 3.39; *p* = 0.0007).

**Figure 2:**
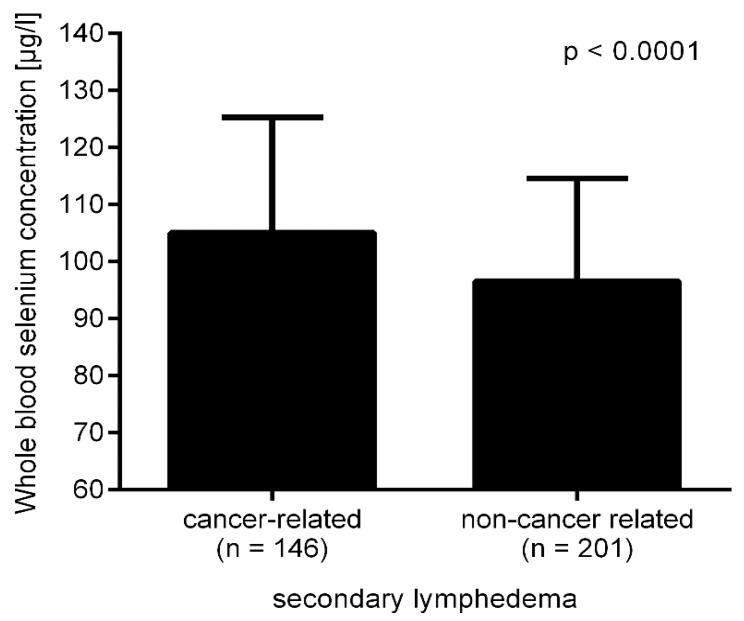
Lower selenium levels in non-cancer related secondary lymphedema.

There was a significant difference in selenium levels between lymphedema patients whose lymphedema was sequelae of cancer treatment and non-cancer lymphedema patients (*p* < 0.0001; Figure 2). Interestingly, the difference in whole blood selenium concentration in cancer and non-cancer patients with lymphedema was only significant in the group with a BMI < 30 (106.2 ± 20.0 vs. 98.6 ± 17.7; *p* = 0.0113) (Table S2). The comparison of these two groups for selenium deficiency reflected this result (BMI < 30 cancer vs. non-cancer OR 1.96; 95% CI 1.07 to 3.63; *p* = 0.0266; BMI ≥ 30 cancer vs. non-cancer OR 2.08; 95% CI 0.96 to 4.74; *p* = 0.0750).

Whole blood selenium concentration was inversely correlated with BMI independent of the disease (Table S1). BMI distribution differed between cancer patients and non-cancer patients with lymphedema (BMI < 30: 80.1% vs. 35.8%; *p* < 0.0266). 

### 3.4. Selenium Deficiency in Lipedema and Lipo-Lymphedema

Whole blood selenium concentration was significantly different between lipedema and lipo-lymphedema (*p* = 0.0127) (Table 3). In addition, there was a significant difference between the prevalence of selenium deficiency (lipedema 41.9%, respectively, lipo-lymphedema 53.0%; *p* = 0.0347). There was no difference between selenium levels, respectively selenium deficiency prevalence in lipedema and lipo-lymphedema patients with a BMI < 30. However, the selenium status was significantly lower in lipo-lymphedema with BMI ≥ 30 (*p* = 0.0264; Table S2). In contrast to lymphedema, there was no significant difference in selenium levels depending on BMI in lipedema and lipo-lymphedema (*p* trend = 0.9888, respectively *p* trend = 0.0642). 

### 3.5. Obesity Increases the Risk for Selenium Deficiency in Lymphedema

**Figure 3:**
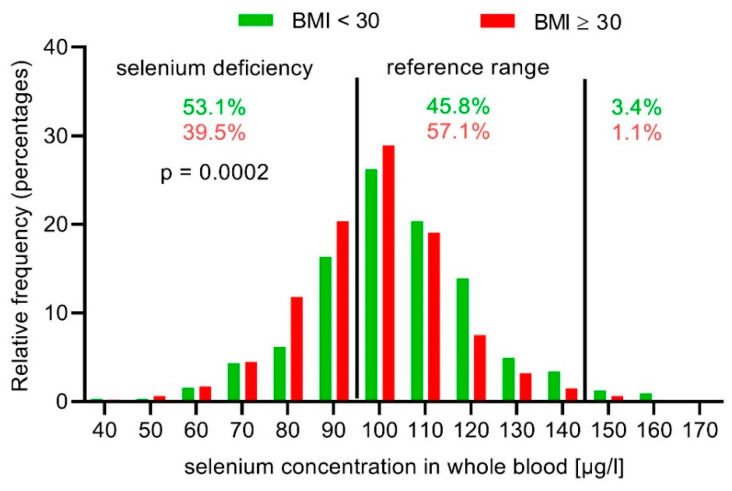
Selenium concentration in whole blood in obese lymphedema, Lipo-lymphedema, and lipedema patients (BMI ≥ 30), compared to normal and overweight patients (BMI < 30).

**Figure 4:**
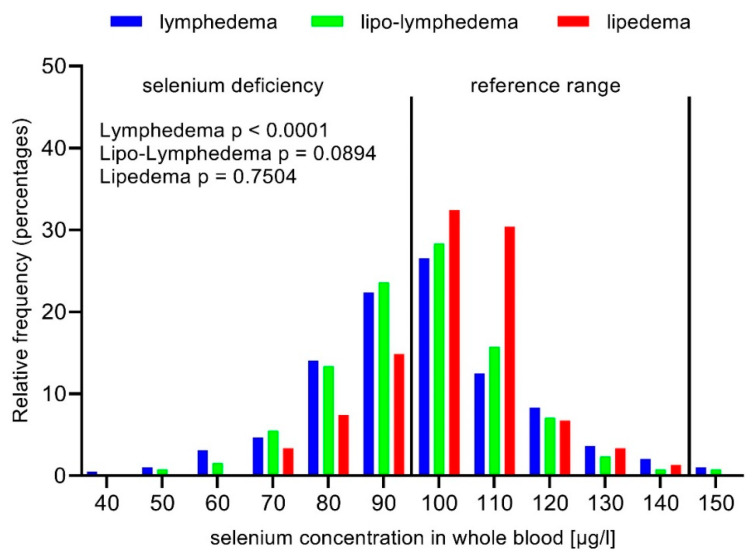
Selenium concentration in whole blood in obese lymphedema, lipo-lymphedema, and lipedema patients (BMI ≥ 30).

Obesity increased the risk of selenium deficiency in the study population (BMI < 30 vs. BMI ≥ 30: 39.5% vs. 53.1%; *p* = 0.0002) (Figure 3). The different diseases showed a more detailed picture. Selenium concentration in whole blood was significantly decreased in obese patients with lymphedema or lipo-lymphedema, but not with lipedema (Table S2; Figure 4). When comparing obese patients with selenium deficiency to patients with a BMI < 30, only obese lymphedema patients displayed a significantly increased risk (OR 2.19; 95% CI 1.49 to 3.21; *p* < 0.0001) (Table 4).

## 4. Discussion

### 4.1. Selenium Deficiency in the Total Study Population

This study aimed to determine the prevalence of selenium deficiency in patients with lymphedema, lipedema, or lipo-lymphedema and a possible influence of obesity as an associated medical condition. The selenium levels in this trial were comparable to whole blood selenium values determined by Muecke et al. in German patients treated in a family doctor practice [35]. Patients without a tumor showed a mean value of 98.9 ± 19.3 μg/L selenium in whole blood. These data are comparable with the values in this study (overall mean selenium concentration 100.6 ± 17.4). The results of Muecke et al. and our results show that the mean selenium levels are slightly below or above the German reference range of whole blood selenium (100–140 μg/L). It is also apparent that selenium levels vary widely, with whole blood selenium concentrations as low as 43.3 μg/L.

Overall, our results showed that selenium deficiency is commonly associated with these medical conditions in a country with low selenium intake. Obesity was an additional risk factor for selenium deficiency in lymphedema.

### 4.2. Selenium Deficiency in Lymphedema

Lymphedemas are divided into primary and secondary (acquired) lymphedema. Primary lymphedema is the result of genetic defects that alter the development of the lymphatic vasculature and typically manifests during infancy, childhood, or adolescence. Less common, primary lymphedema appears after age 35 [36]. Secondary lymphedema is typically caused by surgery, radiation therapy, infection, or trauma. In recent years, another reason is increasingly gaining clinical importance: obesity-related lymphatic impairment [7].

Selenium status was compared in primary and secondary lymphedema. There was no significant difference in selenium levels or selenium deficiency prevalence in the study cohort. Only in secondary lymphedema did selenium levels decrease with increasing BMI, while selenium levels in primary lymphedema were strongly reduced at BMI ≥ 30. There was no further decline in patients with BMI ≥ 40. This probably reflects the different etiologies of primary and secondary lymphedema. Obesity seems to be the main risk factor for selenium deficiency in secondary lymphedema. However, obesity can also be a cause of secondary lymphedema. A reduced selenium status is often associated with increased oxidative stress in several diseases and in obesity. The gradual decline in secondary lymphedema could reflect this increasing oxidative stress [22,37,38]. On the other hand, the relationship between obesity and the lymphatic system is bidirectional, e.g., defects in the lymphatic function also contribute to the development of obesity [36].

Selenium levels decreased and the risk for selenium deficiency increased with increasing lymphedema stage, possibly due to increased oxidative stress. Progression in lymphedema manifests as fibrosis, which is also closely connected to oxidative stress [1,39]. Siems et al. showed that oxidative stress is increased in lymphedema, although there are no data regarding oxidative stress as a function of lymphedema stage [22].

Non-cancer patients with secondary lymphedema displayed significantly lower selenium levels and a higher risk for selenium deficiency compared to the study patients, in whom lymphedema was sequelae of cancer treatment. This result was unexpected, as cancer patients are at risk of selenium deficiency [40,41,42,43,44,45,46]. Therefore, lower selenium levels would be expected in patients with cancer-related lymphedema.

One explanation could be the much higher percentage of obese patients with non-cancer-related lymphedema compared to patients with cancer-related lymphedema. Yet, selenium levels were only non-significantly lower in both obese groups. The significant difference in selenium status was observed in normal weight and overweight patients. If obesity was excluded, one would expect lower selenium levels in cancer patients, not in non-cancer patients, as cancer is also associated with oxidative stress [39]. Siems et al. showed that oxidative stress was increased in chronic lymphedema, but was comparably lower in healthy controls and patients after cancer treatment without lymphedema [22]. Therefore, additional oxidative stress due to cancer or cancer treatment does not seem to increase the risk for selenium deficiency in lymphedema patients. At present, there is no explanation for the increased selenium deficiency risk in non-cancer patients compared to cancer patients.

In summary, selenium deficiency appears to be common in German lymphedema patients. There are some trials that investigated the effect of the inorganic selenium form, sodium selenite, in cancer related lymphedema [17,18,47,48,49]. In these trials, the combination of high-dosed sodium selenite and standard treatment significantly improved lymphedema compared to standard treatment alone. Those trials did not investigate whether the positive effect was dependent on selenium status. Han et al. treated selenium-replete lymphedema patients only and showed a positive effect of sodium selenite [50]. Additionally, sodium selenite was used in many intensive care trials to correct selenium deficiency [51,52,53,54,55].

The effect of selenium deficiency on lymphedema development and progression has not been investigated to date. Avraham et al. showed that Th2 differentiation is essential for the development of lymphatic dysfunction and that inhibiting Th2 cell differentiation with IL-4 and IL-13 monoclonal antibodies improved lymphatic function and decreased fibrosis [56]. Additionally, Th2 helper cells are required for the development of lymphedema and Th2 cytokines; IL-4, and IL-13 are anti-lymphangiogenic, adding to the pathophysiology of lymphedema [1,57]. Selenium deficiency favors Th2 differentiation [58]. Therefore, selenium deficiency might increase the potential for lymphedema as an additional risk factor. TGF-β is significantly increased in lymphedema and plays an essential role in the development of fibrosis, a hallmark of lymphedema progression [1]. Selenium deficiency causes thyroid fibrosis, which is involved in the pathogenesis of myxoedematous cretinism [59]. TGF-β plays a key role in this process. Oglio et al. showed that TGF-β disrupted the redox balance in thyroid cells and increased reactive oxygen species [60]. Selenium might partially reverse the effect of TGF-β. Thus, selenium deficiency appears to play a role in lymphedema progression. 

### 4.3. Obesity Drives Selenium Deficiency

The relationship between obesity and the lymphatic system is bidirectional, e.g., defects in lymphatic function contribute to the development of obesity [36]. Conversely, obesity can result in lymphatic impairment [7]. Additionally, obesity is a major risk factor for the development of breast cancer-related upper extremity lymphedema [42]. 

Obesity was prevalent in the study population, especially in lipedema and lipo-lymphedema patients. Selenium levels decreased in obese patients independent of the medical condition. The risk for selenium deficiency increased 1.7-fold in obese patients. This effect on selenium status was observed in primary and secondary lymphedema, as well as in lipo-lymphedema. 

This is consistent with the results of several trials. Alasfar et al. showed that selenium levels were significantly decreased in morbidly obese females [12]. Additionally, selenium status was decreased in obese children [61,62]. In French women, obesity was associated with decreased selenium levels [10]. In a systematic review, Hosseini et al. concluded that the lower status of antioxidants, i.e., selenium, appears to be associated with obesity [9].

On the other hand, lipedema selenium levels remained stable independent of BMI. Oxidative stress plays a role in both lymphedema and lipedema [22,23,63]. In obesity and lymphedema, there are changes in the immune response [7,56]. The overnutrition-like status of adipocytes dictates changes in adipose immune cells’ composition and causes chronic low-grade inflammation, similar to that found in chronic lymphedema [49]. So far, there is no evidence for an altered immune response or inflammation in lipedema. Under conditions of selenium deficiency, innate and adaptive immune responses are impaired [64]. Inflammation is associated with decreased selenium levels [15]. Therefore, one factor for the higher risk of selenium deficiency in lymphedema and lipo-lymphedema could be chronic inflammation.

### 4.4. Differences in Selenium Status Between Lipedema and Lymphedema

The pathogenesis of lymphedema is described as a progressive process, consisting of lymphatic leakage and stagnation, chronic inflammation, adipose tissue expansion and fibrosis [1]. In contrast to lymphedema, lipedema is characterized by the bilateral increased circumference of extremities, pain sensations, and bruising [2]. The combination of lipedema and lymphatic impairment is called lipo-lymphedema [6].

Interestingly, the different etiology of lymphedema and lipedema seems to be reflected by the selenium status. In contrast to lymphedema, lipedema did not display decreasing selenium levels with increasing stage (data not shown). Progression in lymphedema is associated with fibrosis, which is closely connected to oxidative stress [1,39]. In lipedema, progression is characterized by increased adipose tissue and liposclerosis [6]. Siems et al. detected high baseline levels of both malondialdehyde and protein carbonyls in lipedema, which are indicative of severe pre-existing oxidative stress and likely represent an accelerated lipid peroxidation in lipedematous tissue [23]. There are no published data comparing oxidative stress levels in lymphedema and lipedema. Therefore, it is speculative whether progressing lymphedema accelerates oxidative stress, thereby leading to an increased selenium requirement and by implication reduced selenium levels, while oxidative stress in lipedema is increased but stable.

Neither did selenium status decrease in lipedema with increasing BMI in contrast to lymphedema. Lipo-lymphedema displayed a trend of decreasing selenium levels with increasing BMI, which is consistent with its description as a lipedema with lymphatic changes. One possible explanation could be the definition of obesity. It is long known that lipedema do not react to diets or exercise. Lipedema are often misdiagnosed as obesity. However, lipedema is a disorder of the adipose tissue [65]. Therefore, it could be an explanation that part of the weight gain in lipedema is different from “normal” weight gain. Then, processes such as increased oxidative stress or chronic inflammation behind obesity that seem to be associated with the decrease in selenium levels would play a bigger role in lymphedema, compared to lipedema or lipo-lymphedema.

Obesity had a significant effect on selenium status in lymphedema and lipo-lymphedema, but not in lipedema. Obesity itself showed a significant effect on selenium levels. A comparable number of lipedema and lipo-lymphedema patients in this study were obese. Only obese lymphedema and lipo-lymphedema patients, not obese lipedema patients, displayed a significant decrease in selenium concentration in whole blood compared to normal- and overweight patients. Therefore, there seems to be an additional factor in lymphedema development and/or progression, which results in decreased selenium levels. So far, there is only a case report in which selenium, in combination with Butcher’s broom, was used to treat a patient with stage II lipedema and lipo-lymphedema [66]. The combination helped maintain limb volume reduction after complete decongestive therapy.

### 4.5. Strengths and Limitations

The strength of this study is the total number of participants with lymphedema, lipedema, and lipo-lymphedema, as well as reasonable high numbers for the various diseases, respectively their sub-groups. A further strength is the determination of selenium using whole blood, as it is a better indicator for long-term selenium status compared to serum selenium concentrations. Third, all selenium measurements were conducted in the same certified laboratory ensuring that the results were comparable. Fourth, the specialization of the clinic in these diseases and its long-term experience ensured a correct diagnosis and classification.

Limitations: First, the observational design did not allow for causal relationships across variables to be established. Second, the study population was inherently composed of more advanced diseases, given the specialization of the clinic. Third, the data of the study population was collected during two different time periods that differed in length (4 years vs. 2 years) and patient numbers (245 vs. 552). In the first time period, selenium levels were only measured in selected patients, in whom selenium deficiency was deemed probable. In the second time period, selenium status was measured in most clinic patients. Forth, the selenium levels were comparable to German patients treated in a family doctor practice [35]. However, representative data regarding the selenium status in healthy Germans are not available. Therefore, the question remains; if selenium values in lymphedema patients are significantly lower compared to healthy German people.

## 5. Conclusions

In countries with selenium intake below recommendations, selenium deficiency is common in secondary lymphedema and lipo-lymphedema. The risk for low selenium values increases in lymphedema patients, if they are also obese. A low selenium status could have a negative effect on secondary lymphedema and lipo-lymphedema, as selenium deficiency is associated with an impaired immune function. Further research and clinical trials are necessary to gain more insight into whether selenium deficiency is cause or result, especially in secondary lymphedema. Nonetheless, it would be sensible to check the selenium status in lymphedema patients, especially if they are obese, as infection risk is already increased in lymphedema. 

## Figures and Tables

**Table 1:** Descriptive characteristics of patients.

Characteristic	*n* = (%) *
Sample, *n*	791
Primary lymphedema	78
Secondary lymphedema, total	347
Non-cancer	201
Cancer	146
Lipedema	198
Lipo-lymphedema	168
Selenium status ^†^, μg/L, mean (SD)	100.6 ± 17.4
Selenium deficiency	376 (47.5)

* Unless otherwise specified; ^†^ Selenium concentration in whole blood.

**Table 2:** Selenium concentration in lymphedema.

Characteristic	Selenium Concentration in Whole Blood *, μg/L, Mean (SD)	
	Primary Lymphedema	Secondary Lymphedema
Sample, *n*	78	347
All	104.0 ± 20.9	100.5 ± 20.3
Selenium deficiency, *n* (%)	34 (43.6)	170 (49.0)
BMI < 30	108.8 ± 22.2	103.3 ± 19.5
BMI ≥ 30 < 40	97.2 ± 18.2	99.4 ± 22.9
BMI ≥ 40	99.9 ± 15.4	96.0 ± 19.9
*p* trend ^†^	0.0646	0.0041

* Unless otherwise specified; ^†^ *p* trend values were calculated using one-way ANOVA for. *p* < 0.05 are printed in bold letters.

**Table 3:** Selenium concentration in whole blood in lipedema and lipo-lymphedema.

Characteristic	Selenium Concentration in Whole Blood *, μg/L, Mean (SD)		
	Lipedema	Lipo-Lymphedema	*p* Value ^†^
Sample, *n*	198	168	
All	101.7 ± 12.3	98.4 ± 15.6	0.0127
Selenium deficiency, *n* (%)	83 (41.9)	89 (53.0)	0.0347
BMI < 30	101.7 ± 14.2	103.3 ± 13.3	0.7343
BMI ≥ 30 < 40	101.8 ± 11.1	97.7 ± 18.9	0.0692
BMI ≥ 40	101.5 ± 14.0	96.4 ± 14.4	0.0254

* Unless otherwise specified; ^†^ *p* values were calculated using *χ*^2^ test for categorical variables and Student’s t test or Mann–Whitney test. *p* < 0.05 are in bold letters.

**Table 4:** Odds ratio for selenium deficiency in patients with lymphedema, lipo-lymphedema, and lipedema.

	Odds Ratio (95 % CI)	*p* Value ^†^
Total		
BMI < 30	Ref.	Ref.
BMI ≥ 30	1.73 (1.30 to 2.30)	0.0002
BMI ≥ 40	1.76 (1.27 to 2.43)	0.0006
Lymphedema		
BMI < 30	Ref.	Ref.
BMI ≥ 30	2.19 (1.49 to 3.21)	<0.0001
BMI ≥ 40	2.37 (1.49 to 3.74)	0.0002
Lipo-Lymphedema		
BMI < 30	Ref.	Ref.
BMI ≥ 30	1.85 (0.90 to 3.71)	0.0894
BMI ≥ 40	1.88 (0.91 to 4.11)	0.0995
Lipedema		
BMI < 30	Ref.	Ref.
BMI ≥ 30	0.95 (0.46 to 1.93)	0.8811
BMI ≥ 40	1.11 (0.58 to 2.11)	0.7504

^†^*p* values were calculated using *χ*^2^ test. *p* < 0.05 are printed bold.

## Supplementary Materials

The following are available online at https://www.mdpi.com/2072-6643/12/5/1211/s1, Table S1: Descriptive characteristics of patients stratified for BMI. Table S2: Selenium concentration in obese and morbidly obese patients with lymphedema, lipo-lymphedema, and lipedema. Table S3: Obesity in lymphedema, lipo-lymphedema, and lipedema in two time periods.
