# Supplementary material for: Selenium Deficiency in Lymphedema and Lipedema—A Retrospective Cross-Sectional Study from a Specialized Clinic
Source: Nutrients. 2020 Apr 25;12(5):1211. doi: 10.3390/nu12051211 (PMC7281982; doi:10.3390/nu12051211)
Supplement: Supplementary file 1 [file nutrients-12-01211-s001.pdf]

Table S1. Descriptive characteristics of patients stratified for BMI.

**Characteristic**	**n = (%)***			
	**BMI < 30**	**BMI ≥ 30 < 40**	BMI ≥ 40	All, n
Sample, n	324	196	271	791
Primary lymphedema	44 (56.4)	26 (33.3)	8 (10.3)	78
Secondary lymphedema	189 (54.5)	54 (15.5)	104 (30.0)	347
Non-cancer	72 (35.8)	37 (18.4)	92 (45.8)	201
Cancer	117 (80.1)	17 (11.6)	12 (8.2)	146
Lipedema	50 (25.2)	73 (36.9)	75 (37.9)	198
Lipo-lymphedema	41 (24.4)	43 (25.6)	84 (50.0)	168
Selenium status†, µg/l, mean (SD)	103.8 ± 18.5	99.1 ± 15.6‡	97.8 ± 16.7‡	100.6 ± 17.4
Selenium deficiency	128 (39.5%)	103 (52.6%)‡	145 (53.5%)‡	376 (47.5)

*Unless otherwise specified; † selenium concentration in whole blood; ‡ $p < 0.05$ compared to BMI < 30; p values were calculated using χ^2 test for categorical variables and Student's t test or Mann-Whitney test for continuous variables. SD, standard deviation

Table S2. Selenium concentration in obese and morbidly obese patients with lymphedema, lipo-lymphedema, and lipedema.

**Characteristic**	**Selenium concentration in whole blood*, µg/l, mean (SD)**		
	BMI < 30	BMI ≥ 30	p value†
Sample, n	324	467	
All	103.8 ± 18.5	98.3 ± 16.3	**< 0.0001**
Selenium deficiency, n (%)	128 (39.5%)	248 (53.1%)	**0.0002**
Lymphedema	104.3 ± 20.1‡	96.7 ± 18.5‡	**< 0.0001**
Secondary lymphedema	103.3 ± 19.5	96.4 ± 18.7	**0.0006**
Primary lymphedema	108.8 ± 22.2	97.8 ± 17.4	**0.0068**
Lipo-lymphedema	103.3 ± 13.3‡	96.8 ± 16.0‡	**0.0264**
Lipedema	101.7 ± 14.2‡	101.7 ± 12.6‡	0.8305
p trend†	0.6484‡	**0.0093**‡	
Secondary lymphedema			
Cancer (n = 146)	106.2 ± 20.0	100.9 ± 21.1	0.2342
Non-cancer (n = 201)	98.6 ± 17.7	95.5 ± 18.1	0.1583
p value †	**0.0113**	0.1362	
Selenium deficiency, n (%)			
Lymphedema	91 (39.5)	113 (58.9)	**< 0.0001**
Lipo-lymphedema	17 (41.5)	72 (56.7)	0.0894
Lipedema	20 (40.0)	63 (42.6)	0.7504

*Unless otherwise specified; † p values were calculated using χ^2 test for categorical variables and Student's t test or Mann-Whitney test for continuous variables and ordinary one-way ANOVA for p trend, respectively. $p < 0.05$ are in bold letters. ‡ These values were used to calculate the p-trend values.

Table S3. Obesity in lymphedema, lipo-lymphedema, and lipedema in two time periods.

Characteristic	No. n (%)*		
	2012 - 2016	2018 - 2019	p value†
Sample, n	236	555	
BMI < 30	156 (66.1)	168 (30.3)	**< 0.0001**
BMI ≥ 30	80 (33.9)	387 (69.7)	
Lymphedema			
BMI < 30	113 (81.3)	120 (42.0)	**< 0.0001**
BMI ≥ 30	26 (18.7)	166 (58.0)	
Lipo-lymphedema			
BMI < 30	25 (35.2)	16 (16.5)	**0.0053**
BMI ≥ 30	46 (64.8)	81 (83.5)	
Lipedema			
BMI < 30	18 (69.2)	32 (18.6)	**< 0.0001**
BMI ≥ 30	8 (26.7)	140 (81.4)	
Secondary lymphedema			
Cancer (n = 146)			
BMI < 30	50 (96.2)	67 (71.3)	**0.0003**
BMI ≥ 30	2 (3.7)	27 (28.7)	
Non-cancer (n = 201)			
BMI < 30	42 (70.0)	30 (21.3)	**< 0.0001**
BMI ≥ 30	18 (30.0)	111 (78.7)	

*Unless otherwise specified; † p values were calculated using χ^2 test for categorical variables. $p < 0.05$ are in bold letters.
